# Macrophage contributes to radiation-induced anti-tumor abscopal effect on transplanted breast cancer by HMGB1/TNF-α signaling factors

**DOI:** 10.7150/ijbs.57445

**Published:** 2021-03-01

**Authors:** Lin Zhu, Songling Hu, Qianping Chen, Haowen Zhang, Jiamei Fu, Yuchuan Zhou, Yang Bai, Yan Pan, Chunlin Shao

**Affiliations:** 1 Institute of Radiation Medicine, Shanghai Medical College, Fudan University, Shanghai 200032, China.; 2 State Key Laboratory of Radiation Medicine and Protection, School of Radiation Medicine and Protection, Medical College of Soochow University, Suzhou 215123, China.; 3 Department of Radiation Oncology, Shanghai Pulmonary Hospital, School of Medicine, Tongji University, Shanghai 200433, China.

**Keywords:** Radiation-induced abscopal effect, Breast cancer cells, Macrophages, HMGB1, TNF-α.

## Abstract

**Objectives:** The roles of innate immunity including macrophages in radiation-induced abscopal effect (RIAE) are ambiguous. In this study, we evaluated the role of macrophage in RIAE and the interaction of cytokines in tumor microenvironment after irradiation.

**Materials and Methods:** Transplanted tumor of breast cancer cells in BalB/C mice, severe combined immunodeficiency (SCID) mice and non-obese diabetic (NOD)-SCID mice were irradiated with fractionation doses to observe anti-tumor abscopal effect. The underlying mechanism of RIAE was investigated by treating the mice with TNF-α inhibitor or macrophage depletion drug and analyzing the alteration of macrophage distribution in tumors. A co-culture system of breast cancer cells and macrophages was applied to disclose the signaling factors and related pathways involved in the RIAE.

**Results:** The growth of nonirradiated tumor was effectively suppressed in mice with normal or infused macrophages but not in mice with insufficiency/depletion of macrophage or TNF-α inhibition, where M1-macrophage was mainly involved. Investigation of the bystander signaling factors *in vitro* demonstrated that HMGB1 released from irradiated breast cancer cells promoted bystander macrophages to secret TNF-α through TLR-4 pathway and further inhibited the proliferation and migration of non-irradiated cancer cells by PI3K-p110γ suppression.

**Conclusions:** HMGB1 and TNF-α contributes to M1-macrophages facilitated systemic anti-tumor abscopal response triggered by radiotherapy in breast cancer, indicating that the combination of immunotherapy and radiotherapy may has important implication in enhancing the efficiency of tumor treatment.

## Introduction

As an important tumor treatment method, radiotherapy (RT) has been employed to about 60% of newly-diagnosed cancer patients 1, 2. In recent decades, many studies have demonstrated that radiation could trigger a series of systemic responses out of the radiation field. RT-induced systemic anti-tumor effect on distal nonirradiated tumors has been termed as abscopal effect 3 that was first introduced by Mole in 1953 4. Local RT could induce abscopal effects in several types of cancers including melanoma, lymphoma and renal-cell carcinoma 5.

Breast cancer is a common disease worldwide 6. Recently, some case reports presented that anti-tumor radiation-induced abscopal effect (RIAE) occurred in breast cancer patients without any combination therapy 7, 8, and preclinical studies revealed the dependence of RIAE on T cells 9. Immune checkpoint inhibitors (e.g., anti-PD-1 agents: pembrolizumab and nivolumab) are effective in enhancing the activity of tumor-reactive T cells in approximately 1/5 to 1/4 of patients with metastatic melanoma, lung cancer and renal carcinoma 10-12. Implications of ipilimumab, an inhibitor of cytotoxic T-lymphocyte antigen 4 (CTLA-4), in tumor radiotherapy have been discussed in the case reports of melanoma 5 and non-small cell lung cancer 13. However, the majority of patients have no effective response to the combination of immunotherapy and radiotherapy (CIR), which may due to the suboptimal radiation doses, the absence of tumor-reactive T cells, and the disturbed immunoregulatory mechanisms 9,14. Obviously, the approaches of CIR based on T cells activation are not perfect for all cancer patients, and other immune cells might motivate an effective anti-tumor abscopal effect as well. It is a significance issue to looking for mechanisms that do not depend on T cells in the anti-tumor effect.

Using *in vitro* cell models, evidence has demonstrated that the pro-inflammatory and immunologic responses are involved in radiation-induced bystander effect (RIBE) that is another concept of RIAE in cell level. It was reported that the cytokines released from irradiated cells contributed to the injuries of bystander cells 15-17. As an intermediary of RIBE, macrophages amplified the bystander responses by releasing much more signaling factors such as tumor necrosis factor (TNF)-α and interleukin (IL)-1α which contributed to the incidence of secondary bystander effect 18. Therefore, we speculate that the innate immune cells (especially macrophages) may contribute to RIAE *in vivo.*

To date, major mechanistic studies of RIAE focus on the role of tumor-reactive T cells. Currently, we investigated the contribution of innate immune system in RIAE of breast cancer cells (BCCs) by using mouse models *in vivo* and cell co-culture system *in vitro*. The tumor growths and macrophage composition and protein expression in primary and abscopal tumors were compared in different mouse models after fractional RT on the primary tumor, and the abscopal signaling factors was further investigated by using an* in vitro* cell co-culture system containing BCCs and activated macrophages.

## Methods and Materials

### Animal models

Six-week-old female BalB/C mice, severe combined immunodeficiency (SCID) mice and non-obese diabetic (NOD)-SCID mice were purchased from Shanghai Lab Animal Resource Center (STCSM, Shanghai, China). SCID mice are born without functional B-and T-cells due to gene mutation 19 but they retain normal innate immune system with functional macrophages, neutrophils and natural killer (NK) cells 20. Besides the B-and T-cell deficiencies, NOD-SCID mice have a weakened function of macrophages and NK cells and are absent of complement activity 21.

These animals (ages of 6-8 weeks and weight of 20-25g) were housed in ventilated cages at 22-24°C and 50-60% humidity in a 12-h light/dark cycling room of the animal facilities in Fudan University and Soochow University. All animal experiments were subject to approval by the Institutional Animal Care and Use Committee at Fudan University and Soochow University, and all experimental procedures are in accordance with the ARRIVE guidelines.

### Cell culture

Mouse breast cancer cell line 4T1, human breast cancer cell line MDA-MB-231, and human promonocytic macrophage cell line U937 were obtained from Shanghai Cell Bank (Chinese Academy of Science, Shanghai, China) and authenticated by Short Tandem Repeat (STR) analysis (Genesky Biotechnologies Inc., Shanghai). Separation and harvesting of mouse peritoneal macrophages (PMφ) are described below. 4T1, PMφ and U937 cells were cultured in RPMI 1640 (Gibco Invitrogen, Grand Island, NY, USA), and MDA-MB-231 cells were cultured in DMEM (Gibco) in an atmosphere of 5% CO_2_ at 37°C. The media were supplemented with 10% fetal calf serum (FCS, Gibco) and 1% penicillin-streptomycin (Gibco).

### Syngeneic or xenograft tumour models

BalB/C mice were inoculated with 4T1 cells. NOD-SCID mice were inoculated with MDA-MB-231 cells. SCID mice were inoculated with either 4T1 cells (group SCID(4T1)) or MDA-MB-231 cells (group SCID(MDA-MB-231)). For the inoculation, 1×10^6^ 4T1 cells were subcutaneously injected into the mammary fat pad at the right flank (termed as primary tumor, Pt) on day 0, and 5×10^5^ cells were injected into the mammary fat pad at the left flank (termed as secondary tumor, St) on day 2. Similarly, 5×10^6^ and 3×10^6^ MDA-MB-231 cells were injected into mammary fat pads to form Pt and St, respectively. In order to reduce the total tumor burden, the number of tumor cells seeded to St was half of Pt. When secondary tumors grew to a specified size (3mm diameter), the mice bearing tumors of similar sizes were randomly divided into control (Sham) and RT group. The maximal and perpendicular diameters of tumors were measured every 3 days using a Vernier caliper and the tumor volume was calculated as (length (mm) × (width (mm))^2^/2. When the volume of secondary tumor approached to 50-100 mm^3^, mice were anesthetized with an intraperitoneal injection of ketamine (100 mg/kg) for irradiation treatment. At 7- and 18-day post-radiotherapy (dpr) for BalB/C mice, and 12-dpr for SCID and NOD-SCID mice. All animals were anesthetized with ketamine and sacrificed by cervical dislocation (Supplementary Fig. S1).

### Fractionated radiotherapy

The primary tumor in mice was irradiated with fractionation doses of 8 Gy X-rays (X-RAD 320, North Branford, CT, USA) per day in three successive days (total dose: 24 Gy) at a dose rate of 2.4 Gy/min (Supplementary Fig. S1A, B). The mouse body beyond of tumor in the RT group and the whole body in the Sham group was well protected by a lead shield with a thickness more than 10mm.

### Macrophage depletion

Liposome-encapsulated clodronate (Clo) (FormuMax Scientifc Inc., Palo Alto, USA) was used to deplete macrophages in SCID and NOD-SCID mice. Mice were administered an intravenous injection with 0.2 ml/mouse of either clodronate-containing liposomes (Clo group) or empty liposomes (Lip group) as a control of clodronate approximately 48 h before tumor cell inoculation so that macrophages were depleted as described previously 22.

### Peritoneal macrophage extraction and reinfusion

Peritoneal macrophages were separated from 8-week healthy female BalB/C mice as described previously 23. To characterize the purity of isolated macrophages, cells were examined after 8 h of isolation by flow cytometry (described below). Tumor cells were inoculated on both sides of NOD-SCID mice with depleting macrophages. After fractionation RT of primary tumor, NOD-SCID mice were infused with 1×10^6^ peritoneal macrophages or its PBS control through the tail vein once a day for 3 days.

### Flow cytometry assay of peritoneal macrophages

1×10^6^ extracted peritoneal macrophages were analyzed by flow cytometry according to the method described before 23. Briefly, cells were stained with APC-conjugated anti-mouse F4/80 antibody (mAbs) (eBioscience, San Diego, CA, USA) and resuspended for FACS assay (FACSan Gallios, Beckman, USA).

### *In vivo* TNF-α inhibition

Lenalidomide (Celgene, Summit, NJ, USA), an inhibitor of TNF-α 24, was dissolved in dimethyl sulfoxide (DMSO) and diluted with phosphate buffered saline (PBS) before use. It was administered (50 mg/kg) intraperitoneally every day after RT until sacrificed to inhibit TNF-α production in SCID mice. The control group was injected with PBS containing the equivalent concentration of DMSO.

### Bouin's fixative straining

Mouse lung tissues were fixed for 24 h in Bouin's fixative and soaked with anhydrous alcohol to restore the color of lung tissue. The white nodules of metastatic breast cancer on pulmonary surface were observed and counted under an anatomical microscope.

### Tumor immunohistochemistry (IHC) and immunofluorescence (IF)

Breast cancer tumors were embedded in paraffin for IHC and IF assay. The tumor sections were incubated with primary antibodies for 12 h at 4 °C. For IHC assay, the applied antibodies were anti-PI3-kinase p110γ, anti-CD56 (Santa Cruz Biotechnology Inc., Dallas, TX, USA; 1:200), anti-CD68, anti-TNF-α (Abcam, Cambridge, MA, USA; 1:200), anti-F4/80 (Cell Signaling Technology, Inc., Danvers, MA, USA; 1:200), anti-E-Cadherin (HuaAn Biotechnology Co. Ltd., Hangzhou, China; 1:200), and corresponding HRP-labeled secondary antibodies. HRP staining was visualized with DAB Kit (Dako, Glostrup, Denmark). The positive color was brown. For IF assay, F4/80^+^/iNOS^+^ and F4/80^+^/CD206^+^ macrophages were identified with primary antibodies of anti-F4/80 (Cell Signaling Technology, 1:200), anti-iNOS and anti-CD206 (Abcam, 1:200). Each tissue section was photographed in at least 6 fields randomly and analyzed with the ImageJ software (National Institutes of Health, USA). The average optical density of positive areas in each field was counted from 10 independent tumor sections. Quantification of immunostaining densities were analyzed by the color deconvolution method using Image J software (NIH, USA).

### Cell co-culture

U937 is usually applied to study macrophage differentiation and activation. To differentiate into macrophage-like cells, U937 cells were treated with 50 ng/ml phorbol-12-myristate 13-acetate (PMA, Sigma-Aldrich, St. Louis, MO, USA) for 24 h. After PMA treatment, U937 becomes adherent with an increased expression of macrophage surface antigen CD68 25. BCCs were irradiated with 4 Gy γ-rays at a dose rate of 0.73 Gy/min using a ^137^Cs irradiator (Gammacell 40, MDS Nordion International Inc., Ontario, Canada). Then macrophages (PMφ or U937) were co-cultured with the same amount of irradiated or non-irradiated BCCs for 24 h, and the medium of these co-cultured cells was collected as conditioned medium (CM). Subsequently, other BCCs were treated with CM for 24 h. The outline of this process was illustrated in the supplementary Fig. 1c.

### Cell treatment

BCCs were treated with 400 pg/mL of TNF-α (Beyotime Biotech Ltd. Haimen, China) for 24 h and then their proliferation and migration were detected. U937 cells were treated with recombinant human high-mobility group box 1 (HMGB1) (Beyotime) for 24 h and then the expression of TNF-α and TLR-4 was detected. In the co-culture system containing U937 cells and irradiated BCCs, anti-human TNF-α antibody (1 μg/mL, R&D Systems, Inc. Minneapolis, MN, USA) was added to the medium during 24 h of cell co-culture to neutralize the secreted TNF-α.

### Cell proliferation assay

Cell proliferation was measured using a Cell Counting Kit-8 reagent (CCK-8, Dojindo, Kumamoto, Japan). After each treatment, 4T1 and MDA-MB-231 cells (3×10^3^ per well) were seeded into 96-well plates and incubated for 3 days in a humidified incubator. Then 10 μL of CCK-8 reagent was added to each well. After 30 min, the reaction was stopped by DMSO. The optical density at 450 nm was measured using a microplate reader (Tecan infinite M200 pro, Männedorf, Switzerland) to evaluate the cell proliferation rate.

### Wound healing and transwell assays for cell migration

4T1 cells (4×10^5^ per well) were seeded into 6-well plates. The adherent cells were treated with CM or other indicated reagents for 24 h. The culture was scratched a line using a sterile tip (200 µL) and washed with PBS triply, then incubated in serum-free medium for 24 h. The culture was photographed immediately after 0 h and 24 h of wound formation. Wound areas were measured using ImageJ software. Cell migration rate was calculated as: (wound width (0 h) - wound width (24 h)) / wound width (0 h).

For MDA-MB-231 cell migration assay, after treatment with CM or reagents, 3×10^4^ cells in 400 μL medium were seeded in an insert dish with 8 μm pores (Corning, NY, USA). 0.9 mL medium with 10% FCS was added in the lower chamber of a 24-well plate to encourage cell migration. After 24 h of culture, cells on the lower surface of insert dish were fixed with methanol, stained with crystal violet and counted randomly in six fields (×100) using ImageJ software.

### Western blot assay

Total proteins of cells and tissues were extracted and measured by Western blot assay using primary antibodies of anti-Slug, anti-E-Cadherin, anti-p-Akt (Thr308), anti-Akt (pan), anti-PI3-kinase p110γ (Cell Signaling Technology, 1:1000), anti-TLR4 (Santa Cruz; 1:1000), anti-HMGB1, anti-TNF-α (Proteintech Group, Inc., Rosemont, IL, USA; 1:1000), anti-transferrin (Abcam, 1:1000) and anti-β-actin (Beyotime; 1:1000). Proteins transferred on a PVDF membrane were incubated with primary antibodies overnight at 4°C and labeled with secondary HRP-conjugated antibodies (Beyotime; 1:1000) for 2 h at room temperature. The protein bands were observed using a ChemiDoc XRS system (Bio-Rad Laboratories, Hercules, CA, USA) and analyzed using the Quantity One software (Bio-Rad Laboratories).

### Cytokine assay

To examine cytokine in the cell free supernatant, cell co-culture medium was centrifuged to harvest the supernatant. To examine cytokine in mouse serum, blood was collected and centrifuge to obtain serum. The concentrations of inflammatory factors of TNF-α and IL-10 in the cell co-culture medium and mouse serum were measured with corresponding ELISA kits, including mouse TNF-α ELISA kit, mouse IL-10 ELISA kit, human TNF-α ELISA kit, human IL-10 ELISA kit (Neobioscience Technology Co., Ltd. China), according to the manufacturer's instructions. Each sample was assayed in triplicate for each cytokine together with the positive and negative controls.

### Statistical analysis

All data were obtained from more than three independent experiments and expressed as means ± SD. Comparisons between two groups were performed using an unpaired two-sided Student's t-test for continuous variables or Chi-square test for categorical variables. All statistical analyses were perform using SPSS software (SPSS Inc., Chicago, IL, USA). *P*<0.05 was considered as significant difference between indicated groups.

## Results

### Radiation-induced growth delay of abscopal tumor is associated with the innate immune system

**Figure 1:**
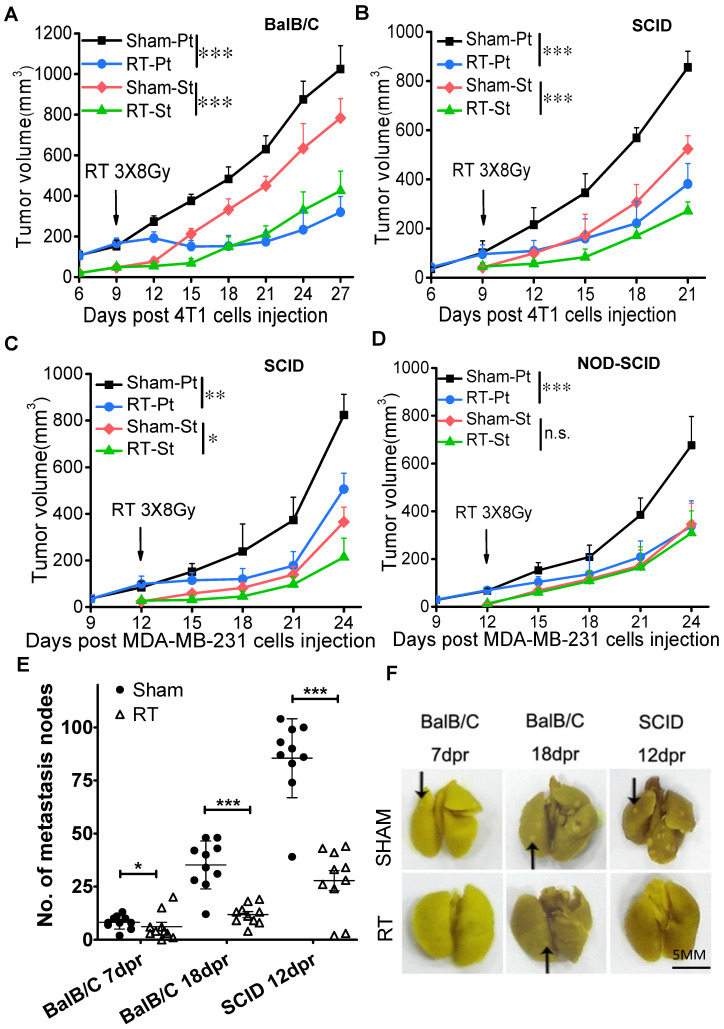
** Fractionation irradiation induces anti-tumor abscopal effect in BalB/C and SCID mice.** The primary and secondary tumors of group Sham and group RT were marked as Sham-Pt, Sham-St, RT-Pt and RT-St, respectively. The growths of both primary and secondary tumors were delayed in radiotherapy (RT) group of BalB/C mice (A), SCID(4T1) (B), SCID(MDA-MB-231) mice (C), but not in NOD-SCID mice (D). (E) Total numbers of nodules on the surface of lung were decreased by RT in BalB/C and SCID mice at 7 dpr, 18dpr (BalB/C) and 12 dpr (SCID), respectively. (F) Representative photographs of mice lung with metastasis nodules of 4T1 cells. Black arrows point to lung nodules. Data are from 10 mice/group (n=10) and are presented as the mean ± SD. * *p*<0.05 and *** *p*<0.001 between indicated groups. n.s. not significant. Scale bar, 5mm.

After fractionation irradiation of primary tumors in three mouse models, the growth of irradiated tumor was inhibited as expected (Fig. 1), and the growth of secondary tumor was also suppressed in BalB/C mice (Fig. 1A, Supplementary Fig. S2A), SCID(4T1) mice (Fig. 1B, Supplementary Fig. S2B) and SCID(MDA-MB-231) mice (Fig. 1C), indicating the occurrence of anti-tumor RIAE. It has been known that T cells contributed to RIAE 9, consistent with our result that the abscopal effect was induced in BalB/C mice that has a normal immune system containing T cells. Interestingly, we found that the anti-tumor RIAE also occurred in the SCID mice that had no functional T cells but still retained innate immune cells. To further verify the requirement of the innate immune system in RIAE, we inoculated MDA-MB-231 cells into NOD-SCID mice which are absent of innate immunity and lymphocytes. It was found that the growth of primary tumor was significantly inhibited by the fractionation irradiation, but the growth of secondary tumor was not reduced in NOD-SCID mice (Fig. 1D). In addition, it was observed that breast cancer 4T1 cells were prone to pulmonary metastasis since the numbers of pulmonary surface nodules were obviously increased in BalB/C and SCID mice without RT (Fig. 1E, F). But when the primary tumor was irradiated with fractional doses, the number of lung nodules was significantly decreased in BalB/C and SCID mice. Therefore, the innate immune cells should participate the incidence of anti-tumor RIAE.

### Effect of radiotherapy on macrophage distribution in tumor tissues

**Figure 2:**
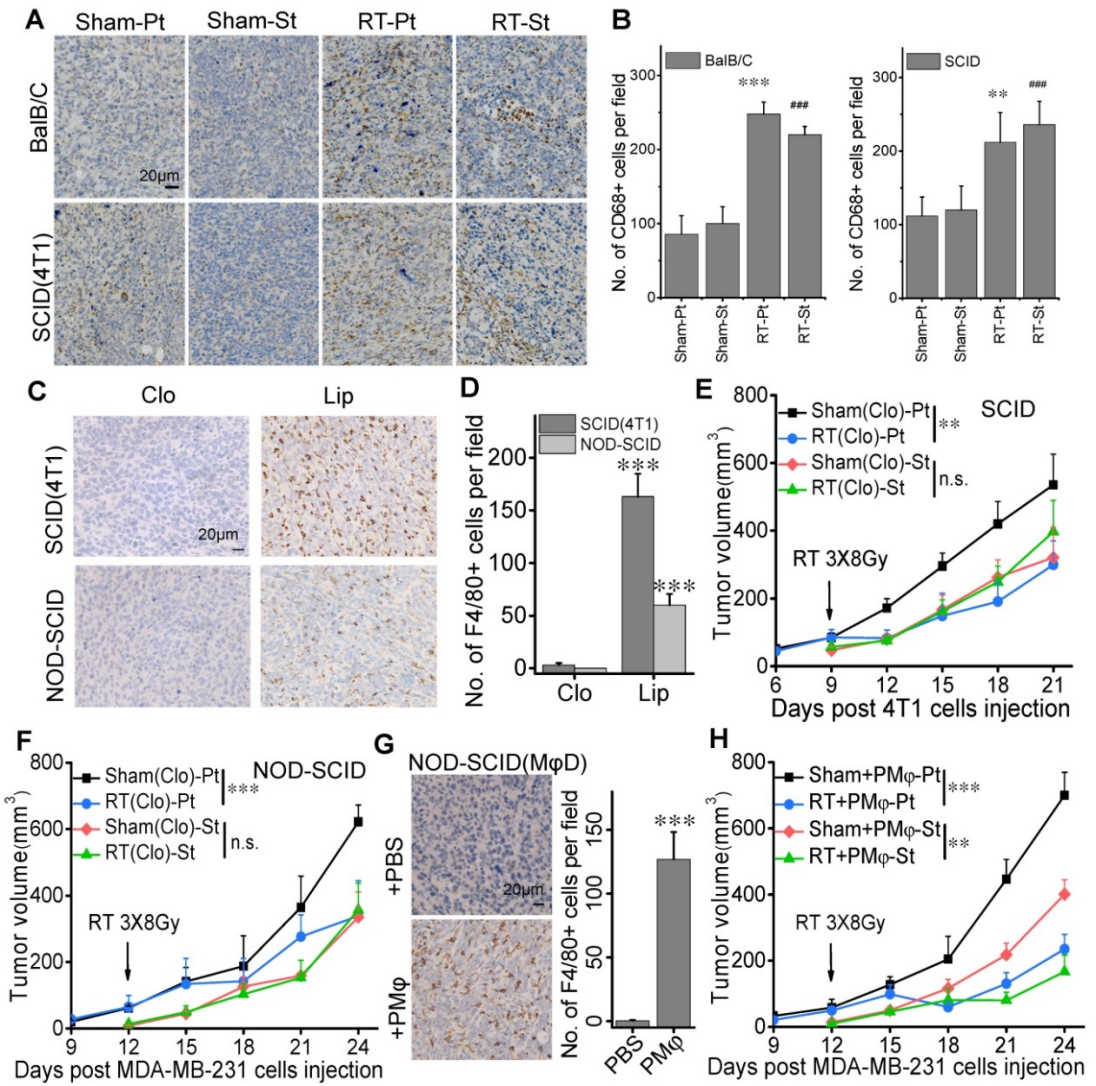
** RIAE was associated with macrophage infiltration in the tumors.** Macrophage distribution was identified by CD68 or F4/80 in the primary and secondary tumors in the groups of Sham-Pt, Sham-St, RT-Pt and RT-St, respectively. Cell nuclei (blue) were counterstained with hematoxylin. (A) Representative image of CD68 (brown). (B) Quantification of immunostaining densities in (A) analyzed with Image J software. ** *p*<0.01 and *** *p*<0.001 versus Sham-Pt. # *p*<0.05, ## *p*<0.01 and ### *p*<0.001 versus Sham-St. (C) Representative images of F4/80 (brown) in the tumors of SCID(4T1) and NOD-SCID mice that were injected with clodronate (Clo) or its liposome control (Lip) before inoculation of breast cancer cells. (D) Numbers of positive cell per field (IHC, ×400) in (C). *** *p*<0.001 versus Clo. Growth curves of primary tumor and secondary tumor in SCID(4T1) (E) and NOD-SCID (F) mice with macrophages depletion(MφD). (G) Representative images and numbers of positive cell per field (IHC, ×400) for F4/80 (brown) in the tumor of macrophage depleted NOD-SCID mice with peritoneal macrophage (PMφ) reinfusion. *** *p*<0.001 versus PBS. (H) Growth of secondary tumor was delayed in the macrophage depleted NOD-SCID mice with PMφ reinfusion after RT. Data from 10 mice/group (n=10) are presented as the mean ± SD. In (E), (F) and (H), ** *p*<0.01, *** *p*<0.001, n.s., not significant. Scale bar, indicated in the images.

Concentrating on the role of innate immune system in RIAE in BalB/C and SCID mice, we examined the expression of CD68 as a marker of macrophages in the tumor tissues by IHC and found that CD68 positive cells went up 2.2-2.88 fold and 1.89-1.97 fold in both irradiated tumor and abscopal tumor in BalB/C and SCID(4T1) mice, respectively (Fig. 2A, B), indicating the aggregation of macrophages in tumors after RT. However, the expression of CD56, a marker of dendritic cells (DCs) /NK cells, had no significant difference among the irradiated primary tumor, nonirradiated abscopal tumor and their sham-IR controls (Supplementary Fig. S3).

To further confirm the role of macrophages in RIAE, we depleted macrophages in SCID and NOD-SCID mice by clodronate liposomes. Fig. 2C-D confirmed that numbers of macrophages labeled with F4/80^+^ were dramatically reduced by the treatment of clodronate in comparison with liposome control in the transplanted tumors (Pt) of both SCID and NOD-SCID mice. It was found that, after the clodronate treatment, the growths of the primary tumors were still significantly inhibited by the fractionation irradiation, but the growths of secondary tumors of 4T1 and MDA-MB-231 cells were not reduced in SCID mice (Fig. 2E, Supplementary Fig. S2C) and NOD-SCID mice (Fig. 2F, Supplementary Fig. S2D), respectively.

Furthermore, the macrophage-depleted NOD-SCID mice bearing transplanted tumors were injected with PMφs in 3 consecutive days after primary tumor irradiation. Flow cytometry assay identified that more than 90% of these PMφs were F4/80+ cells (Supplementary Fig. S4). After PMφ reinfusion, F4/80^+^ positive macrophages in the primary tumor of clodronate-treated NOD-SCID mice were recovered (Fig. 2G), and the growths of both irradiated primary tumor and non-irradiated secondary tumor were inhibited in the macrophage-depleted NOD-SCID mice (Fig. 2H). These results demonstrated that macrophages were required for the induction of anti-tumor abscopal effect of RT.

**Figure 3:**
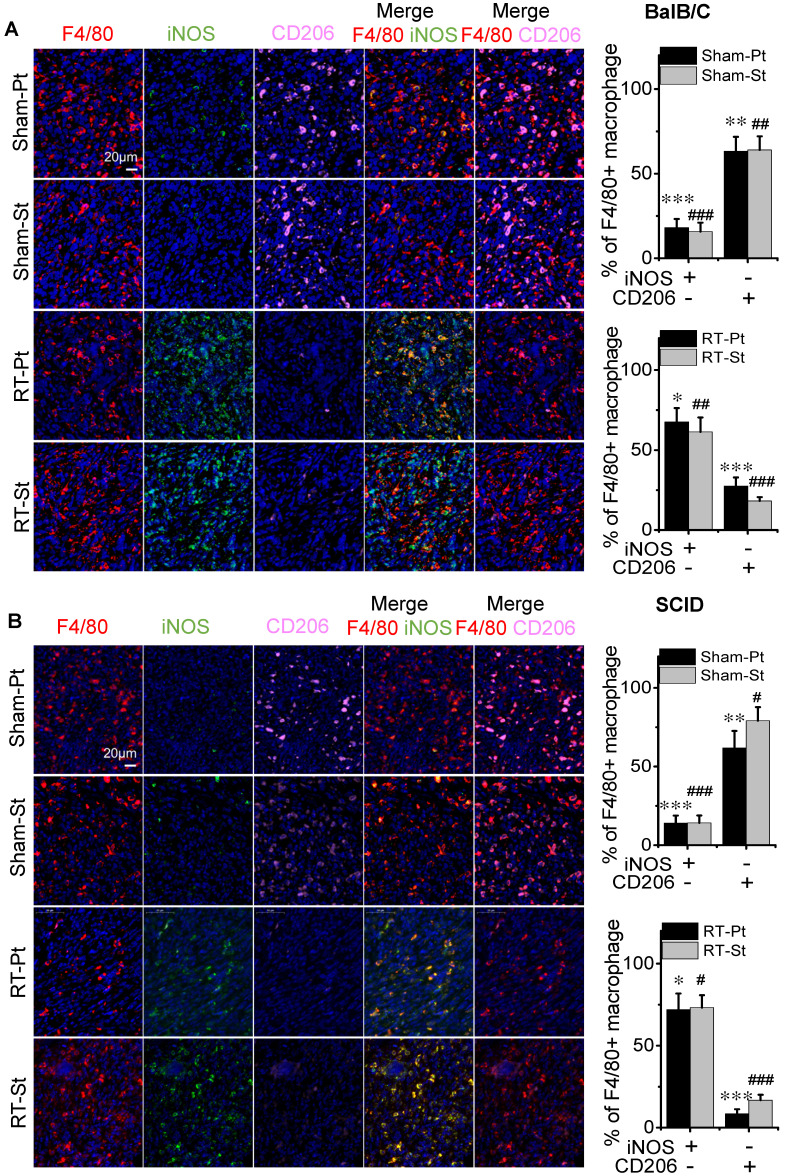
** RT promoted differentiation of macrophages into M1 phenotype.** The primary and secondary tumors of group Sham and group RT were marked as Sham-Pt, Sham-St, RT-Pt and RT-St, respectively. Tumor tissues were stained for F4/80 (red), iNOS (green) and CD206 (pink). Cell nuclei (blue) were counterstained with DAPI. (A) Representative images of tumors in BalB/C mice and the percentages of iNOS^+^CD206^-^ and iNOS^-^CD206^+^ cells in the F4/80^+^ macrophages. (B) Representative images of tumors in SCID (4T1) mice and the percentages of iNOS^+^ CD206^-^ and iNOS^-^CD206^+^ cells in the F4/80^+^ macrophages. Data from 10 mice/group (n=10) are presented as the mean ± SD. * *p*<0.05, ** p<0.01 and *** *p*<0.001 versus F4/80^+^ cells in Pt. # *p*<0.05, ##* p*<0.01 and ### *p*<0.001 versus F4/80^+^ cells in St. Scale bar, 20 μm (×400).

Macrophages are roughly classified into two subtypes termed as classically activated macrophage (M1) and the alternatively activated macrophage (M2) 26. Immunofluorescence staining of F4/80^+^ and iNOS^+^ pointed out the location of M1 macrophages and F4/80^+^ CD206^+^ pointed out the location of M2 macrophages in tumors (Fig. 3). After RT, the percentages of F4/80^+^ iNOS^+^ positive cells in both primary and secondary tumors were increased significantly from 16% of control to 64% in BalB/C mice (Fig. 3A) and from 14% of control to 73% in SCID(4T1) mice (Fig. 3B). Oppositely, after RT, the percentages of F4/80^+^CD206^+^ positive cells in both primary and secondary tumors were significantly decreased from 63% of control to 22% in BalB/C mice (Fig. 3A) and from 70% of control to 13% in SCID(4T1) mice (Fig. 3B). Accordingly, the ratio of M1 macrophage was increased in both irradiated tumor and its abscopal tumor, which may contribute to tumor repression after RT.

### Macrophages suppress cancer cell growth and metastasis

**Figure 4:**
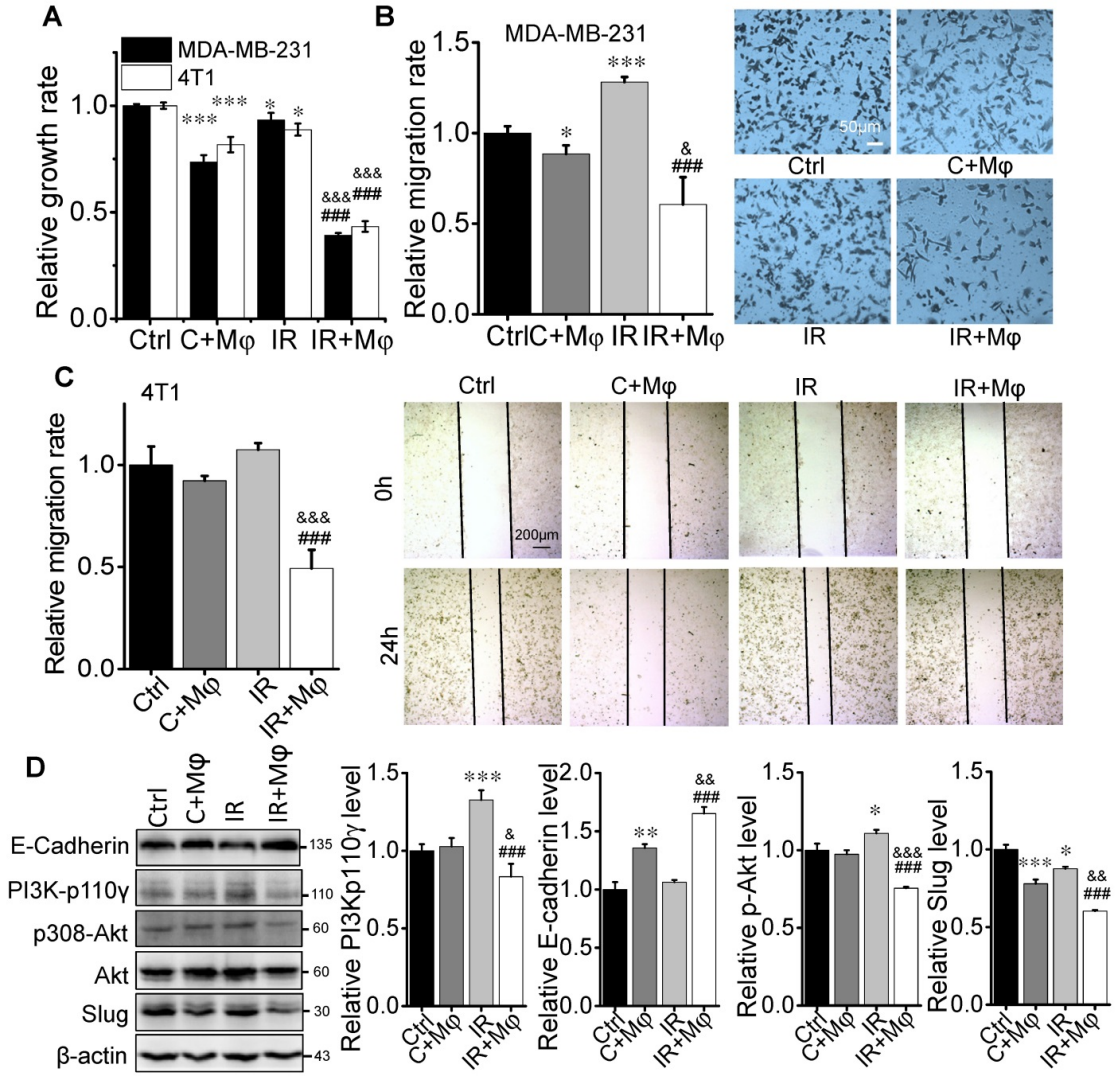
** Macrophage-mediated abscopal effects on proliferation inhibition and mesenchymal-epithelial transition (MET) of breast cancer cells.** The irradiated MDA-MB-231 cells were co-cultured with or without U937 for 24 h. The irradiated 4T1 cells were co-cultured with or without PMφ for 24 h. The conditioned medium (CM) was collected from each group of control (Ctrl), 4 Gy irradiated MDA-MB-231 or 4T1 cells (IR), cancer cells co-cultured with U937 or PMφ, respectively (C+Mφ), and irradiated cells co-cultured with macrophages (IR+Mφ). Then nonirradiated MDA-MB-231 or 4T1 cells were treated with CM from corresponding cancer cells to test the abscopal effects (protocol shown in Fig. S1). (A) Proliferation of abscopal breast cancer cells were determined by CCK-8 assay. (B) Relative migration ability of MDA-MB-231 cells after the treatment of different CM. Scale bar, 50 μm. (C) Relative migration ability of 4T1 cells after the treatment of different CM. Scale bar, 200 μm. (D) Activation of PI3K and MET pathway in abscopal MDA-MB-231 cells and the quantification of relative levels of E-Cadherin, PI3K-p110γ, p308-Akt and Slug normalized to β-actin. Data are presented as the mean ± SD (n ≥ 3). * *p*<0.05, ** *p*<0.01, *** *p*<0.001 versus group Ctrl; & *p*<0.05, && *p*<0.01, &&& *p*<0.001 versus group C+Mφ; ### *p*<0.001 versus group IR.

To have a deep insight into the molecular mechanism of macrophage in RIAE, we applied a cell co-culture system* in vitro* to investigate the interaction between macrophages and BCCs after irradiation. Macrophage-like U937 cells were co-cultured with MDA-MB-231 cells, and PMφ were co-cultured with 4T1 cells. It was reported that fractionated radiotherapy with 3 × 8 Gy would induce systemic anti-tumor responses 27. But this high dose irradiation could cause rapid cell death *in vitro*. Based on our pilot experiment, cells irradiated with 4 Gy, rather than 8 Gy, could release sufficient amount of signaling factors. After 4 Gy irradiation, BCCs were co-cultured with macrophages or cultured alone for 24 h, and the conditional media (CM) from these co-culture cells or from BCCs alone were collected and then was applied to treat the corresponding abscopal BCCs for 24 h (Supplementary Fig. S1 C). The CM was grouped as CM (Ctrl) (the medium of BCCs), CM (C+Mφ) (the medium of BCCs co-cultured with macrophages), CM (IR) (the medium of irradiated BCCs), and CM (IR+Mφ) (the medium of irradiated BCCs co-cultured with macrophages). Correspondingly, the group of abscopal cancer cells treated with above CM was named as Ctrl, C+M, IR and IR+M, respectively. It was found that the proliferation of abscopal cancer cells was repressed by CM (C+Mφ), and particularly repressed by CM (IR+Mφ) (Fig. 4A). The transwell assay showed that, after the CM treatment, the migration of MDA-MB-231 cells was reduced, especially in the group IR+Mφ (Fig. 4B). Similar migration inhibition phenomenon of CM on 4T1 cells was also observed by the wound healing assay (Fig. 4C).

**Figure 5:**
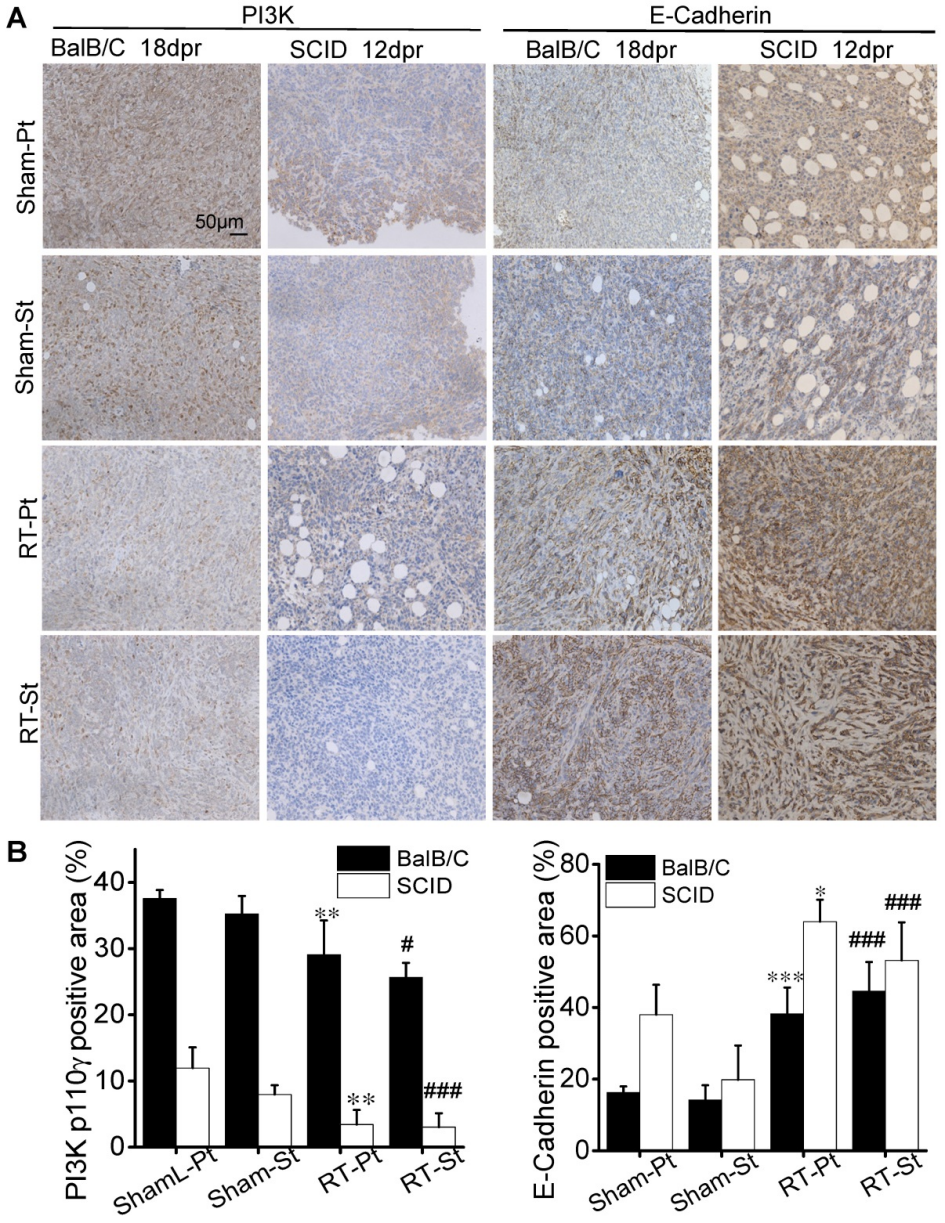
** The effect of RT on the proliferation and migration of cancer cells in tumor tissues.** The primary and secondary tumors in the group Sham and group RT were marked as Sham-Pt, Sham-St, RT-Pt and RT-St, respectively. (A) Representatives of IHC staining for PI3K-p110γ and E-Cadherin (brown) of BalB/C and SCID mice at endpoint. (B) Quantification of the optical densities of the immunostaining area in (A) analyzed by Image J software. Nuclei (blue) were counterstained with hematoxylin (IHC, ×200). Data are from 10 mice/group and are presented as the mean ± SD. * p<0.05, ** p<0.01, *** p<0.001 versus Ctrl-Pt. # p<0.05, ### p<0.001 versus Ctrl-St. Scale bar, 50μm.

Cell migration is closely related to cell growth and transition, thus the cell proliferation associated-pathway of PI3K/Akt and the mesenchymal-epithelial transition (MET)-associated proteins were measured. Fig. 4D illustrates that the expressions of PI3K-p110γ and p-Akt were obviously down-regulated but the expressions of E-Cadherin and Slug were increased in the CM-treated MDA-MB-231 cells of group (C+Mφ) and group (IR+Mφ), especially in the group (IR+Mφ) containing both irradiated cancer cells and macrophages. At the same time, we detected the expressions of E-Cadherin and PI3K-p110γ in the xenograft tumors in BalB/C and SCID(4T1) mice by IHC (Fig. 5). Results also showed that the expression of PI3K-p110γ was reduced but the expression of E-Cadherin was obviously enhanced in both irradiated and abscopal tumors in BalB/C and SCID mice.

The down-regulation of PI3K-p110γ /p-Akt was closely associated with proliferation decline of tumor cells. Up-regulations of E-Cadherin and down-regulation of Slug are critical changes of MET, which means a weakened ability of invasion and migration of cancer cells. These results were consistent with the phenomenon that macrophages in irradiated tumor microenvironment inhibited the growth and metastasis of abscopal cancer cells both *in vitro* and* in vivo*.

### HMGB1 released from irradiated BCCs promotes macrophage differentiation

**Figure 6:**
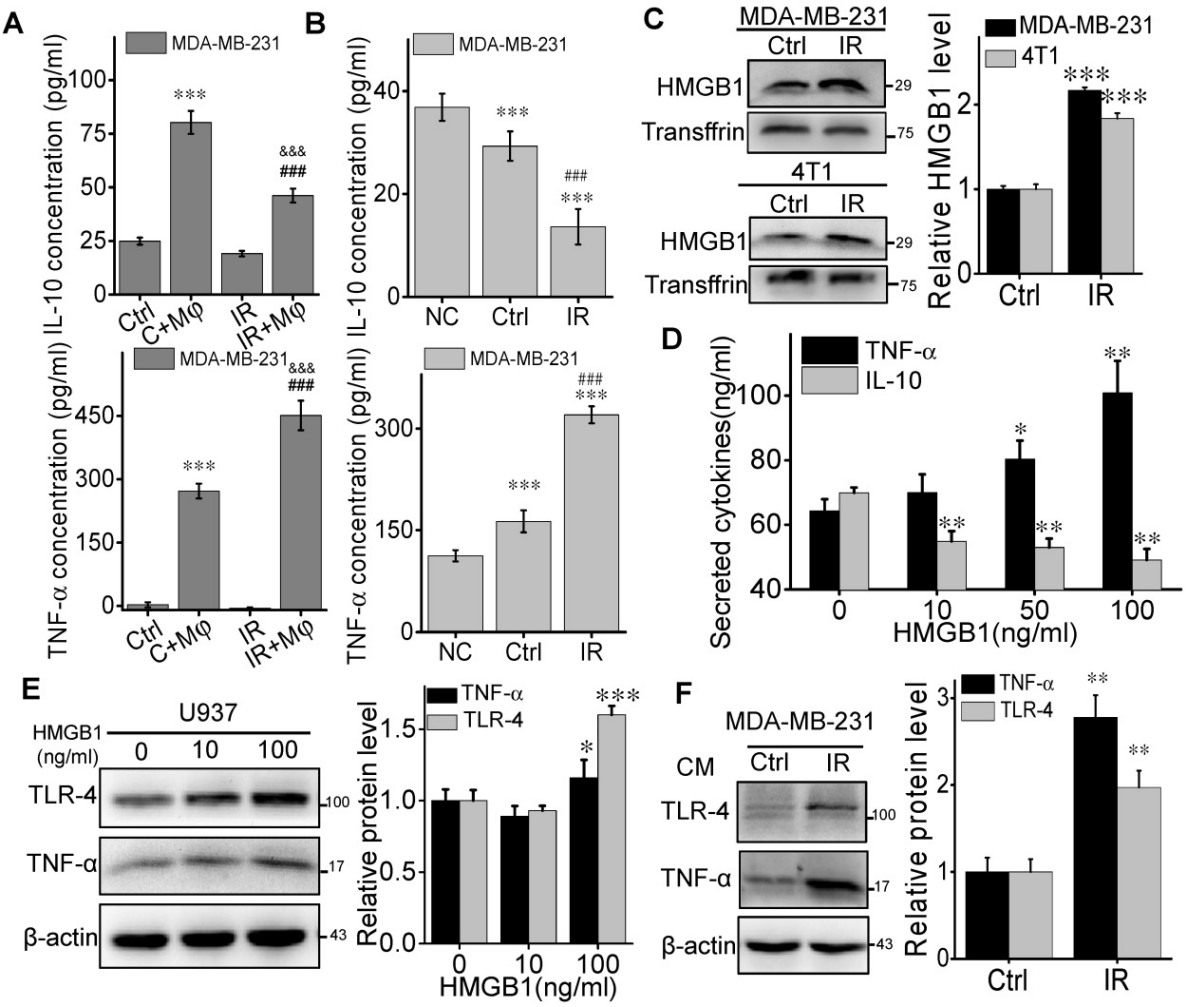
** HMGB1 promoted macrophages to secret TNF-α through TLR-4 pathway.** U937 cells were co-cultured with the same amount of irradiated or non-irradiated MDA-MB-231 cells for 24 h, and the medium of these co-cultured cells was collected as conditioned medium (CM). (A) TNF-α and IL-10 in the CM from co-cultured cells were determined by ELISA kits. *** *p*<0.001 versus CM(Ctrl); &&& *p*<0.001 versus CM (C+Mφ); ### *p*<0.001 versus group IR. (B) U937 cells were treated with DMEM (negative control), the media of MDA-MB-231 cells (Ctrl) and irradiated MDA-MB-231 cells (IR). After 24h, TNF-α and IL-10 in the media from U937 cells were determined by ELISA kits. *** *p*<0.001 versus NC; ### *p*<0.001 versus Ctrl. (C) Western blot assay of HMGB1 in the free-protein obtained from the equal volume media of irradiated 4T1and MDA-MB-231 cells and their control. Quantification of HMGB1 abundance was normalized to Transffrin. *** *p*<0.001 versus Ctrl. (D, E) U937 cells were treated with different concentrations of recombinant human HMGB1 for 24h, then TNF-α and IL-10 in the cell medium were determined by ELISA kits (D) and cellular protein were detected by Western blot assay (E). * *p*<0.05, ** *p*<0.01, *** *p*<0.001 versus group 0. (F) U937 cells were treated with the media of irradiated cancer cells (IR) or its unirradiated control, then cellular proteins were detected by Western blot assay. Quantification of TLR-4 and TNF-α abundance was normalized to β-actin. ** *p*<0.01 versus control group. Data are presented as the mean ± SD (n ≥ 3).

M1 macrophages could secrete high levels of proinflammatory cytokines such as TNF-α and low levels of IL-10, which facilitate a robust anti-tumor activity 28. On the contrary, M2 macrophages have a potential phagocytosis capacity and an anti-inflammatory cytokine profile, which is characterized by a high level of IL-10 production 28. Therefore, to demonstrate the potential signaling factors involved in the macrophage-contributed abscopal response, we investigated the induction of inflammatory factors in the cell co-culture system. ELISA assay demonstrated that the levels of TNF-α and IL-10 in the medium of MDA-MB-231 cells co-cultured with U937 cells were higher than that in the medium of breast cancer cells alone (Fig. 6A). In comparison with CM (C+Mφ), the concentration of TNF-α was significantly elevated while IL-10 was depressed in the CM (IR+Mφ). These data revealed that the irradiated cancer cells promoted the anti-tumor activity and proinflammatory cytokine secretion of macrophages.

To demonstrate where these inflammatory factors are generated from, we sought to split the MDA-MB-231 cell co-culture system by treating U937 cells with DMEM (negative control), CM (Ctrl) or CM (IR) from cancer cells. Then U937 cells were well washed and incubated with fresh DMEM for 24 h, then the conditioned medium was collected for ELISA assay. It was found that, under the situation of irradiation, the concentration of TNF-α in the medium had a much higher level but IL-10 became much lower than those of nonirradiated cancer cells (Fig. 6B). Therefore, the irradiated MDA-MB-231 cells could release some signaling factors that influence the activation of bystander U937 cells, resulting in cytokine alteration in the cell co-culture medium.

We then extracted the proteins in the medium of BCCs at 24 h after 4 Gy irradiation and found that HMGB1 had a high level in both CM of irradiated MDA-MB-231 and 4T1 cells (Fig. 6C). To determine the response of macrophages to HMGB1, we treated U937 cells with different concentrations of HMGB1 from 0 to 100 ng/mL. It was found that, in the CM of U937 cells, TNF-α increased but IL-10 decreased along with the concentration of HMGB1 (Fig. 6D). Meanwhile, the expression of TNF-α and TLR-4 were also increased in the U937 cells after HMGB1 treatment (Fig. 6E). In addition, after treatment with the medium from irradiated MDA-MB-231 cells, U937 cells also expressed high levels of TLR-4 and TNF-α (Fig. 6F). Since the high expression level of TLR-4 is a kind of biomarkers of M1 macrophages 28, HMGB1 released from irradiated BCCs might assist in the anti-tumor activation of macrophages through TLR-4 pathway.

### TNF-α inhibitor eliminates anti-tumor RIAE

**Figure 7:**
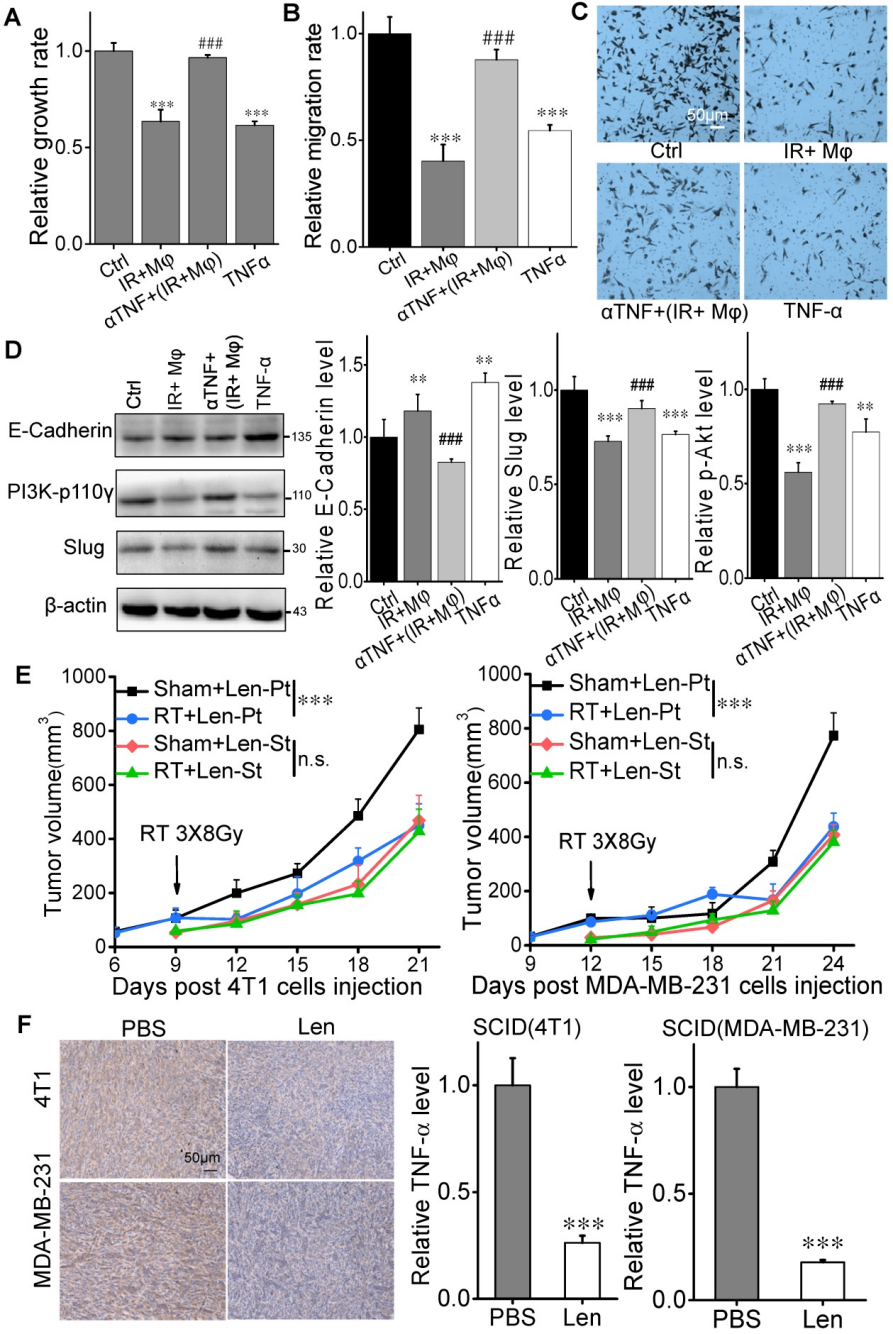
** Irradiation did not induce abscopal effect in TNF-α inhibition models *in vitro* and *in vivo*.** MDA-MB-231 cells were treated with CM (IR+Mφ), CM (IR+Mφ) neutralized by anti-human TNF-α antibody (αTNF + (IR+Mφ)) and TNF-α for 24 h, respectively. CM (IR+Mφ) is the CM from the co-cultures of irradiated cancer cells and U937. (A) Proliferation of breast cancer cells after different treatments. (B) Migration ability of MDA-MB-231 cells after different treatment. (C) Representative images of the above cell migration assay (×200). Scale bar, 50μm. (D) Activation of PI3K pathway and MET in breast cancer cells after different treatments. Western blot analysis was representative of three independent experiments. Data are presented as the mean ± SD (n ≥ 3). * *p*<0.05, ** *p*<0.01 and ***p<0.001 versus control. ## *p*<0.01 and ### *p*<0.001 versus group (IR+Mφ). (E) Tumor growth curves of SCID(4T1) and SCID(MDA-MB-231) mice treated with lenalidomide. F. Inhibitory efficiency of TNF-α in the expression of TNF-α in tumors. Scale bar, 50μm. Data are from 10 mice/group and are presented as the mean ± SD. In (E), ***p<0.001, RT versus sham; n.s. not significant. In (F), *** p<0.001 versus PBS treatment.

To verify whether TNF-α has a direct contribution to the inhibition of tumor cell proliferation and migration, we neutralized the CM of 4 Gy irradiated MDA-MB-231 cells co-cultured with U937 (CM (IR+Mφ)) with anti-TNF-α antibody (αTNF). It was found that this neutralized CM (αTNF + (IR+Mφ)) had no inhibitory effect on the proliferation of MDA-MB-231 cells although CM (IR+Mφ) suppressed cell growth (Fig. 7A). The proliferation of MDA-MB-231 cells was also partly inhibited by the treatment of recombinant human TNF-α. Moreover, the migration of MDA-MB-231 cells was reduced by recombinant human TNF-α and CM (IR+Mφ), which was recovered when CM (IR+Mφ) was neutralized by αTNF (Fig. 7B, C). Consistently, the expressions of PI3K-p110γ and E-cadherin in MDA-MB-231 cells were oppositely changed by the treatment of CM (IR+Mφ) or recombinant human TNF-α, but not by the neutralized CM (αTNF+(IR+Mφ)) (Fig. 7D).

The aforementioned data enlighten that TNF-α might serve as a signaling factor involved in the anti-tumor RIAE. To further verify this speculation, SCID(4T1) and SCID(MDA-MB-231) mice were injected daily with lenalidomide, a selective inhibitor of TNF-α production from monocytes 24, from the day of RT until mice sacrifice. It was found that the growth of primary tumor was still significantly inhibited by the fractionated irradiation, but the growth of secondary tumor was not reduced in these two mouse models (Fig. 7E, Supplementary Fig. S2E, F). Moreover, the TNF-α levels in the primary tumors of both mice were significantly reduced under lenalidomide treatment (Fig. 7F). Taken together, TNF-α produced from macrophages should be an important signaling factor involved in the anti-tumor RIAE.

## Discussion

Radiation-induced abscopal effect is considered as a T cell-dependent immune response. More and more pre-clinical and clinical studies have disclosed that the immune checkpoint inhibitors could considerably alter the treatment efficacy of multiple solid malignancy. However, many tumor patients do not get a positive response and eventually relapse to immune therapy. In melanoma patients who possess a high proportion of tumor-reactive T cells, only 20% of them benefit from this anti-tumor response clinically 29. The reason why only a small part of patients has anti-tumor abscopal response is still elusive 12. On the other hand, studies on T-cell-induced abscopal effect have been mainly performed in normal immune hosts, in which the effect of innate immunity on therapeutics may not be concerned.

Some studies have shown that irradiation promotes anti-inflammatory M2 macrophage phenotype and induces VEGF expression through TNF-α signaling to accelerate tumor regrowth by tumor-associated macrophage (TAM) 30, 31. Opposing results showed that radiation recruits large numbers of myeloid cells to tumor environment in response to immunogenic cell death. Given the increased recruitment of myeloid cells post-RT and the limited efficacy of macrophage targeting alone 32, the myeloid-macrophage compartment makes an ideal target for combining with RT to enhance its anti-tumor efficacy 33. Given the plasticity and diversity of macrophage functions, it is not surprising that irradiation can also program TAMs towards pro-inflammatory phenotype that contributes to anti-tumor response 34. Our current study demonstrated the contribution of M1 macrophages to the anti-tumor RIAE, and the growth of secondary tumor in non-irradiated field was effectively suppressed in BalB/C mice after RT, but there was no RIAE being observed in NOD-SCID and macrophage-depleted SCID mice. However, macrophages attenuated the malignant degree of nonirradiated abscopal tumor cells and played an effective role in RIAE in SCID mice and PMφ re-infused NOD-SCID mice. Therefore, both adaptive and innate immune responses participate in the anti-tumor RIAE.

RT has been proposed to enhance tumor immunogenicity by inducing an immunostimulatory form of cell death and generating inflammatory signals 35. It has been reported that CD47 bind to SIRP-a on macrophage, which is an important mechanism in intervening the capacity of macrophage phagocytosis in irradiated tumor microenvironment (4). Silencing of CD47 and SIRP-a promote breast cancer cells death by PMA-differentiated THP-1 cells (5), and radiation-induced loss of cell surface CD47 enhances immune-mediated clearance of human papillomavirus-positive cancer (6). But the result of phagocytosis could not explain the proliferation inhibition and MET of abscopal cancer cells. On the other hand, released from irradiated cells, the damage-associated molecular patterns (DAMPs) could trigger an adaptive immune response by activating antigen presenting cells (APCs). A prototypic DAMP, HMGB1, closely associated with both acute inflammatory responses and cancer 36, can be released from the dying or stressed cells induced by chemotherapy or radiotherapy 37, 38. DAMPs such as HMGB1 are endogenous TLR activators. TLR4 can be activated by lipopolysaccharide and several DAMP molecules including HMGB1 39. It has been reported that M1 macrophage polarization is related to HMGB1-TLR2/TLR4 cascade signaling 40, and TLR-mediated macrophage activation may result in the localized production of large quantities of TNF-α 41. Our study demonstrated that after 24 h of irradiation, BCCs could release a large amount of HMGB1 that further promoted bystander macrophages to secrete inflammatory factor TNF-α.

Numerous efforts have been performed to investigate the potential signaling factors involved in RIAE. It has been reported that the macrophage derived cytokines (MDC) may induce cellular microenvironment imbalance and trigger radiation-induced systemic effects 42, 43. In a study of abscopal response during RT, the dynamic changes of a panel of 22 cytokines in the patient plasma were analyzed during the course of radiotherapy, where 12 cytokines (eotaxin, IL33, IL6, MCP1, MDC, MIP1α, VEGF, IP10, MCP3, MIP1β, TIMP1, and TNFα) were involved in the intercellular communication and radiation response 43. Our pilot experiments also measured a series of inflammatory factors by ELISA Kits, including TNF-α, TGF-β, IFN-γ, IL-10, IL-6, IL-4, IL-1b, IL-2, IL-18, MMP2, MMP3, MMP8, and granzyme-B in the conditioned cell culture medium, mice serum and breast cancer cell xenograft *in vivo* and found that the cytokine levels of TNF-α and TGF-β were increased but IL-10 was reduced in the irradiated organisms, but the other cytokines had no significantly difference between group control and group RT. Since TGF-β represents an immunosuppressive effect and IL-10 is an iconic inflammatory factor of M2 macrophages 14, we speculated that TNF-α might be an important anti-tumor inflammatory factor in RIAE. This was further verified by using a cell co-culture model and a TNF-α inhibitor injected mouse model.

As a well-known macrophage inflammatory factor, TNF-α is closely related to cell apoptosis. This study indicates that TNF-α has a significant effect on PI3K/Akt pathway. Some other studies suggest that the linkage between TNF and PI3K/Akt may be related to FAK in survival pathway 44-46. It was reported that the Wnt/β-catenin signaling pathway could temporally enhance human ESCs self-renewal by the upregulation of E-cadherin leading to PI3K/Akt activation in embryonic stem cells 47. In contrary, Akt activation could up-regulate the expression of Snail protein and in turn down-regulate the expression of E-Cadherin 48,49, which is consistent with our finding that the E-Cadherin expression was downregulated by the activation of PI3K/Akt and the upregulation of E-Cadherin suppressed Slug expression.

Lenalidomide is one of immunomodulatory drugs (IMiDs) and has been approved for the treatment of multiple myeloma and myelodysplastic syndromes 50. It leads to very potent inhibitor of TNF-α production with anti-proliferative activities against breast cancer cell lines 51. Although exact cellular targets of lenalidomide remain unclear, its anti-inflammatory effect on reducing TNF-α in LPS-stimulated monocytes and co-stimulatory effect on anti-CD3 stimulated T cells were observed 52. Therefore, lenalidomide might not a perfect drug for CIR in breast cancer because of its anti-RIAE effect.

**Figure 8:**
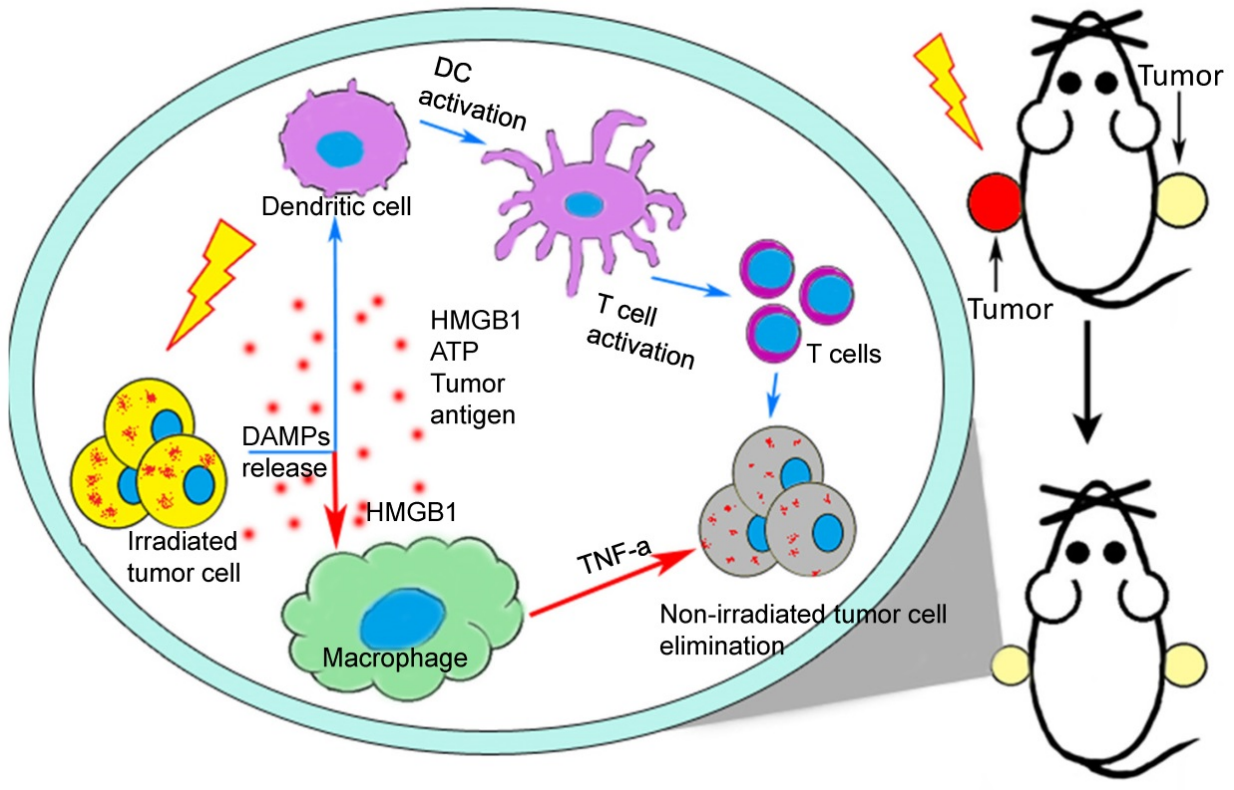
** A proposed model illustrating IR-induced abscopal antitumor effect driven by macrophages and T cells.** After radiotherapy, the irradiated cells release DAMPs to active DC-T cells (blue arrows) 9 and macrophages (red arrows) which would kill tumor cells systematically. HMGB1, one of important DAMPs, promote macrophages to secret TNF-α through TLR-4 pathway and further inhibited the proliferation and migration of non-irradiated cancer cells by PI3K-p110γ suppression.

Over the past half century, the incidence of abscopal effect of conventional RT has been rarely reported. However, with continuous development of targeted immunomodulator, the immune checkpoint blockade and the practice of CIR, the abscopal effect is becoming an increasing significant breakout of tumor therapy. The present study clearly illustrated that radiotherapy-triggered HMGB1 contributed to M1-macrophage-facilitated systemic anti-tumor abscopal response in breast cancer (Fig. 8). It is a great challenge to encourage a systemic anti-tumor immune response by RT. In this respect, the induction of RIAE may have a unique role in arousing immunologic activity systematically.

## Supplementary Material

Supplementary figures.

## Abbreviations

APCs: antigen presenting cells
BCCs: breast cancer cells
CCK: Cell Counting Kit
CIR: combination immunotherapy-radiotherapy
CM: conditioned medium
CTLA: cytotoxic T-lymphocyte antigen
DAMP: damage-associated molecular pattern molecule
DAPI: 4′,6-diamidino-2-phenylindole
DCs: dendritic cells
DMSO: dimethyl sulfoxide
Elisa: enzyme-linked immunoassay
HMGB: high-mobility group box
IF: immunofluorescence
IHC: immunohistochemistry
IL: interleukin
IMiDs: immunomodulatory drugs
IR: irradiation
MDC: macrophage derived cytokines
NK: natural killer
NOD: non-obese diabetic
PBS: phosphate-buffered physiological saline
PMA: phorbol myristate acetate
RIAE: Radiation-induced abscopal effect
RIBE: radiation-induced bystander effect
RT: radiotherapy
SCID: severe combined immunodeficiency
TLR: toll-like receptor
TNF-α: tumor necrosis factor-α
