# Supplementary material for: Macrophage contributes to radiation-induced anti-tumor abscopal effect on transplanted breast cancer by HMGB1/TNF-α signaling factors
Source: Int J Biol Sci. 2021 Mar 1;17(4):926–41. doi: 10.7150/ijbs.57445 (PMC8040298; doi:10.7150/ijbs.57445)
Supplement: Supplementary file 1 — Supplementary figures. [file ijbsv17p0926s1.pdf]

## Supplemental Figures

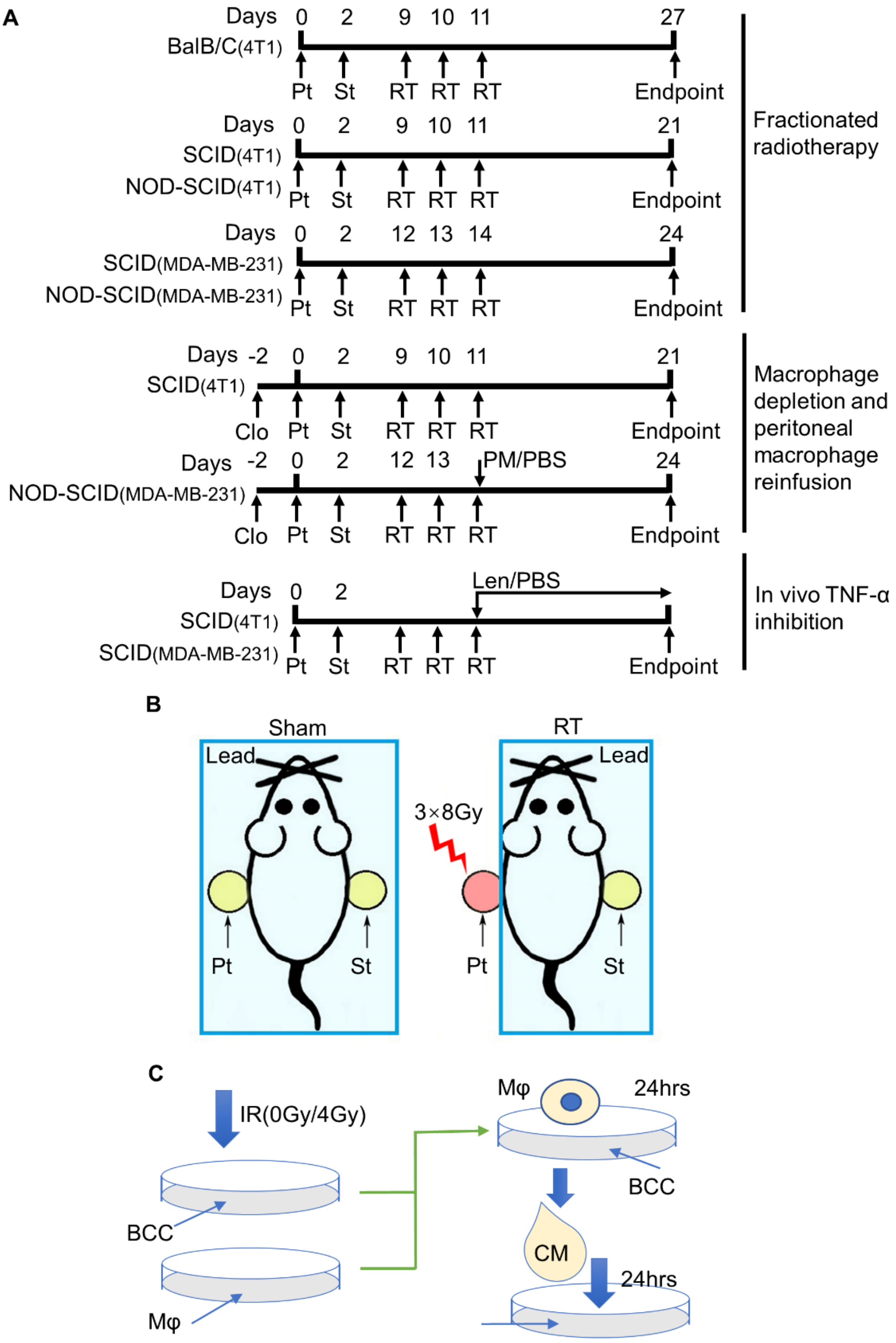


**Figure S1. Models of abscopal effect study.** (A) Timelines of tumor treatment of BalB/C, SCID and NOD-SCID mice. Mice were randomly divided into two groups of Sham and RT based on the similar size of primary tumor (Pt). Fractionated radiotherapy (RT, 8 Gy×3) was only given to the Pt. At the endpoint times (tumor volume less than 1.5 $cm^3$), mice were scarified for further

analysis. Macrophage depletion and peritoneal macrophage reinfusion: mice were administered an intravenous injection with 0.2 ml/mouse of either clodronate-containing liposomes (Clo group) or empty liposomes (Lip group) as a control of clodronate approximately 48 h before tumor cell inoculation. In vivo TNF-α inhibition: lenalidomide was administered (50 mg/kg) intraperitoneally every day after RT until sacrificed to inhibit TNF-α production in SCID mice. (B) Diagrammatic drawing of the irradiation setup. A couple of tumor-bearing mice were placed inside of lead boxes closely. Mice body was protected by lead except for the primary tumor of RT group. (C) Cell co-culture model. 4T1 and MDA-MB-231 cells were irradiated with 4 Gy of γ-rays. Mouse peritoneal macrophages (PMφ) were identified by FACs and co-cultured with 4T1 cells. U937 cells were treated with PMA to differentiate into macrophage-like cells and co-cultured with MDA-MB-231 cells. Macrophages (Mφ) were co-cultured with the same amount of irradiated or nonirradiated homologous breast cancer cells (BCCs) for 24 h. Then the conditioned medium (CM) of the co-cultured cells was collected and applied to treat other untreated BCCs (abscopal cells) for 24 h.

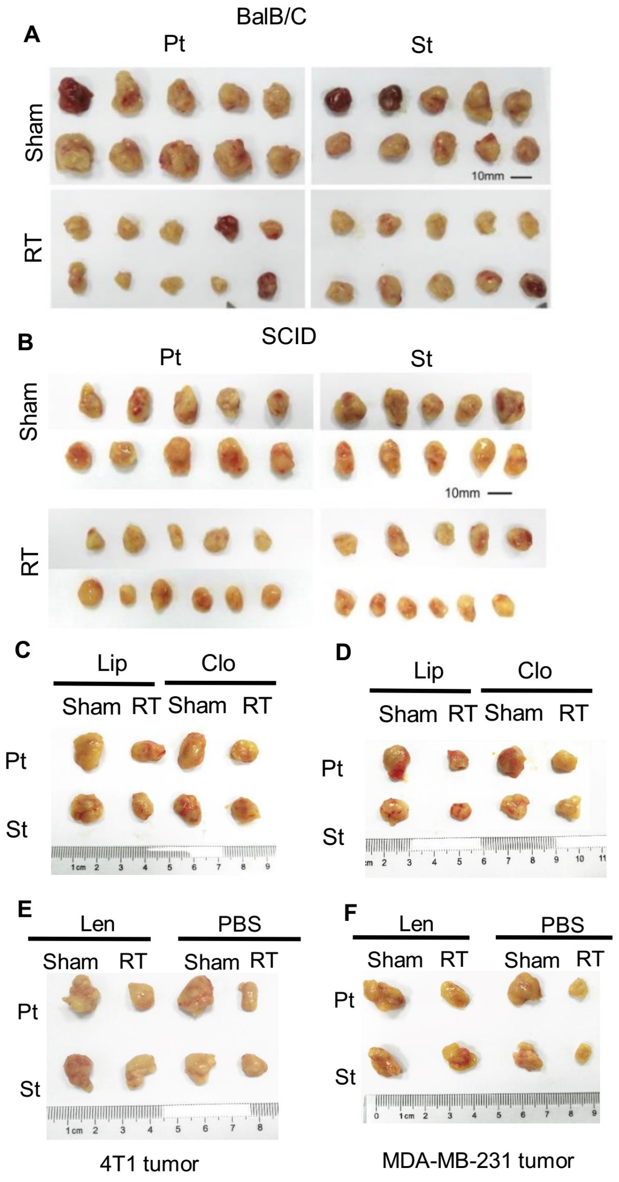


**Figure S2. Representative images of transplanted tumors in BalB/C, SCID and NOD-SCID mice.** The primary and secondary tumors in the group Sham and group RT were marked as Sham-Pt, Sham-St, RT-Pt and RT-St, respectively. The growths of both primary and secondary tumors were delayed in radiotherapy (RT) group of BalB/C mice (A), SCID(4T1) (B). Fractionated radiation on primary tumor (Pt) did not induce abscopal effect on the secondary tumor (St) in SCID and NOD-SCID mice treated with clodronate (Clo) to deplete macrophages before 4T1 cells being inoculated into SCID mice (C) and MDA-MB-231 cells being inoculated into NOD-SCID mice (D). (E) Representative photographs of primary and secondary tumors of SCID(4T1) mice. SCID(4T1) and SCID(MDA-MB-231) mice were injected with PBS or lenalidomide (Len). (F) Representative photographs of primary and secondary tumors of SCID(MB231) mice.

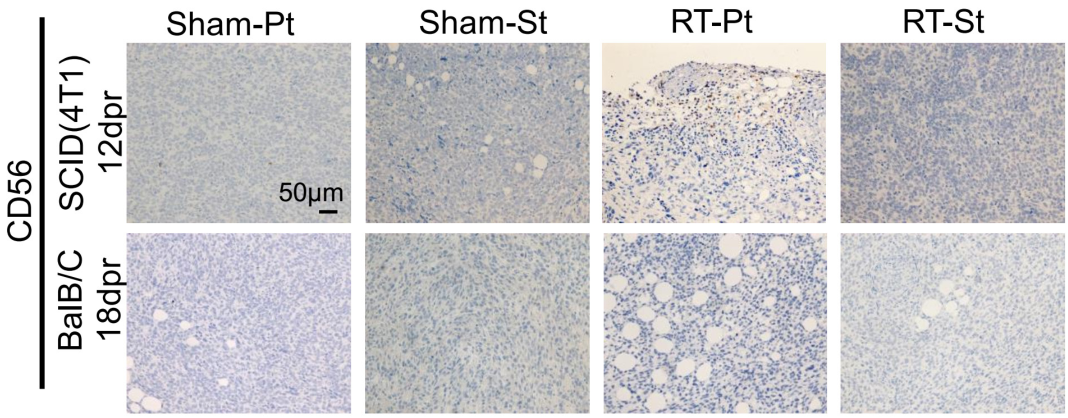


**Figure S3. RT did not affect $CD56^+$ cells infiltration in the tumors.** The primary and secondary tumors in the group Sham and group RT were marked as Sham-Pt, Sham-St, RT-Pt and RT-St, respectively. Cell nuclei (blue) were counterstained with hematoxylin (IHC, ×200). Representative image of immunohistochemical staining for CD56-positive cells (brown). Scale bar, 50 μm.

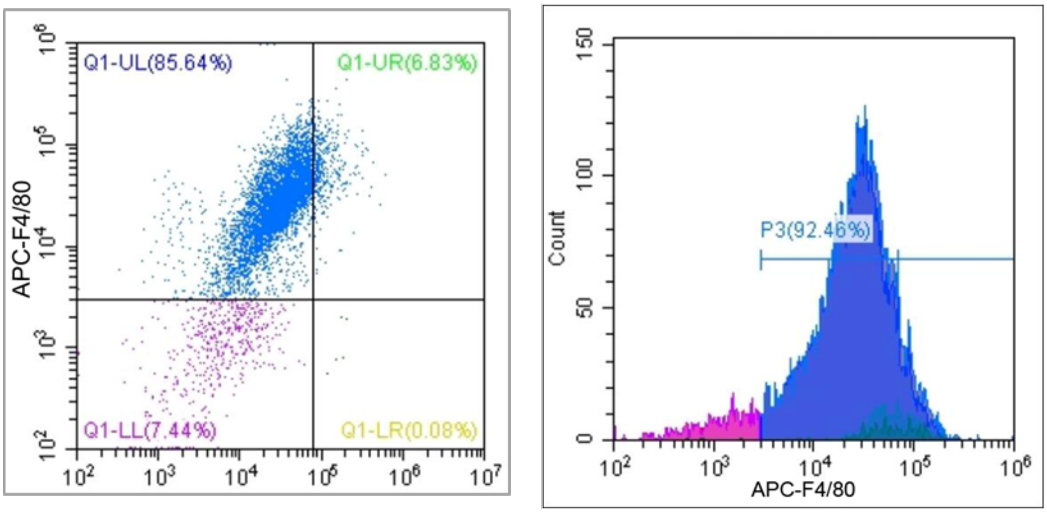


**Figure S4. Verification of the peritoneal macrophages obtained from 8-week healthy female BalB/C mice.** The expression of F4/80 positive cells was examined by flow cytometry.
